# Diagnostic Value of Non-Contrast CT in Cerebrospinal Fluid Leakage After Endoscopic Transnasal Surgery for Sellar and Suprasellar Tumors

**DOI:** 10.3389/fonc.2021.735778

**Published:** 2022-01-20

**Authors:** Wei Gao, Xiaoyu Wang, Yuanjian Fang, Yuan Hong, Wei Yan, Sheng Zhang, Chenguang Li

**Affiliations:** ^1^ Department of Neurosurgery, The Second Affiliated Hospital of Zhejiang University, Hangzhou, China; ^2^ Department of Neurosurgery, Changxing People’s Hospital, Changxing, China; ^3^ Department of Neurology, Zhejiang Provincial People’s Hospital, People’s Hospital of Hangzhou Medical College, Hangzhou, China

**Keywords:** cerebrospinal fluid leakage, endoscopic transnasal surgery, head CT, pneumocephalus, diagnosis

## Abstract

We aimed to study the relationship between pneumocephalus on non-contrast CT (NCCT) and post-operative cerebrospinal fluid leakage (p-CFL) after endoscopic transsphenoidal sellar and suprasellar tumor surgeries. Data from patients who underwent endoscopic treatment for sellar or suprasellar tumors from January 2018 to March 2020 were consecutively collected and reviewed. The NCCT pneumocephalus (NP) was measured the first day after operation and the first day after the expansive sponge was extracted. p-CFL was determined according to post-operative clinical symptoms, high resolution CT and glucose test, and expert consensus. Of the 253 patients enrolled in this study, 32 (12.6%) had p-CFL. Compared with patients without p-CFL, patients with p-CFL had a higher occurrence of intra-operative CFL, a longer operation time, a higher rate of pneumocephalus on first-day NCCT after operation (i.e., first-day NP), and a higher rate of NP volume change between two NCCT measurements (referred to as the NP change) (all *p* < 0.05). In multivariate regression analysis, first-day NP was independently associated with p-CFL occurrence [odds ratio (OR)=6.395, 95% confidence interval (CI)=2.236-18.290, p=0.001). After adding the NP change into the regression model, first-day NP was no longer independently associated with p-CFL, and NP change (OR = 19.457, 95% CI = 6.095–62.107, p<0.001) was independently associated with p-CFL. The receiver operating characteristic curve comparison analysis showed that NP change had a significantly better predicting value than first-day NP (area under the curve: 0.988 vs. 0.642, Z=6.451, p=0.001). NP is an effective imaging marker for predicting p-CFL after endoscopic sellar and suprasellar tumors operation, and the NP change has a better predicting value.

## Introduction

Endoscopic transsphenoidal surgery is increasingly performed by neurosurgeons to treat skull base lesions, but cerebrospinal fluid (CSF) leakage (CFL) is a difficult-to-avoid complication, with an incidence as high as 11% (1). The most common sites of surgical traumatic CFL are the ethmoid roof and sphenoid sinus (2). In one study, pituitary tumor resections accounted for nearly half of the cases of confirmed CFL following tumor removal (3). CFL can cause symptoms of low intracranial pressure, pneumocephalus, and life-threatening intracranial infection, all of which seriously affect patient prognosis (4, 5). Although there are many ways to repair CFL, such as lumbar cistern drainage and multilayered techniques including fat tamponade, fascia lata, artificial dura, pediculate nasoseptal flap, and balloon compression (6–10), leakage is still difficult to repair. Moreover, post-operative CFL is difficult to detect and easily neglected by clinicians.

Early detection of CFL is quite important. Methods reported to diagnose CFL include β2‐transferrin testing, glucose rhinorrhea content analysis, high-resolution computed tomography (HRCT), magnetic resonance imaging (MRI), and cisternography (11–13). However, these methods are inconvenient and carry a certain risk of misdiagnosis (14, 15). Pneumocephalus is a common clinical manifestation of CFL (16). In cases of low intracranial pressure, air can enter the brain and cause pneumocephalus (17, 18). Non-contrast CT (NCCT) can clearly show pneumocephalus after transsphenoidal surgery. However, since no study has assessed the relationship between pneumocephalus on NCCT and CFL after transnasal surgery, the diagnostic value of post-operative NCCT for CFL is uncertain.

Here, we analyzed surgical cases of endoscopic transsphenoidal sellar and suprasellar tumors, focusing on the relationship between CFL and the occurrence and volume change of NCCT pneumocephalus (NP) to identify a new method of CFL diagnosis.

## Methods

### Ethics Statement

Each subject or an appropriate family member provided written informed consent prior to the study, and the protocols were approved by the local ethics committee. All clinical investigations were conducted according to the principles expressed in the Declaration of Helsinki.

### Study Subjects

**Figure 1:**
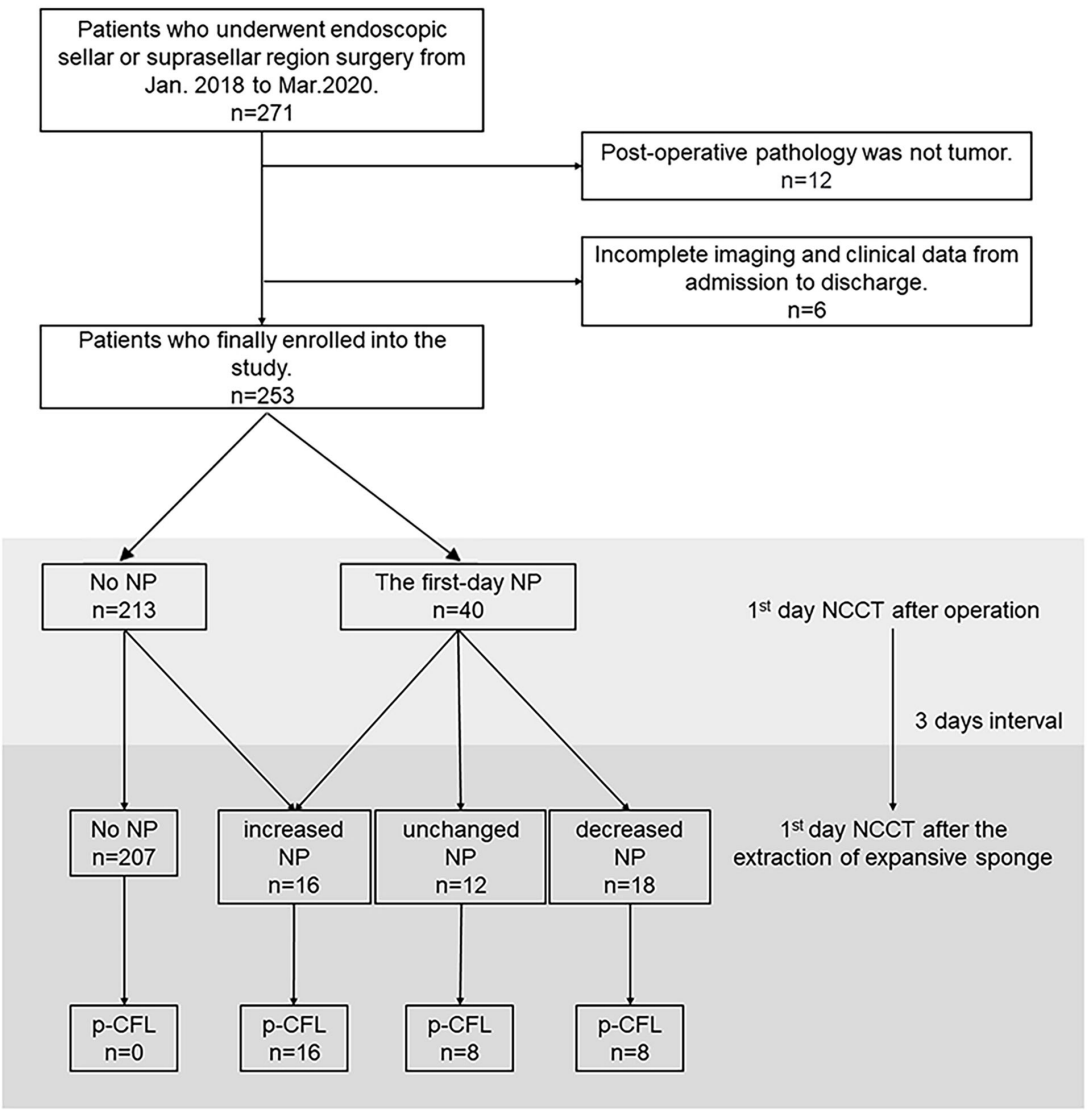
Flow chart depicting patient selection and the observation process for the association between NP and p-CFL. NCCT, non-contrast computed tomography; NP, NCCT pneumocephalus; p-CFL, post-operative cerebrospinal fluid leakage.

The retrospective study included consecutively collected patients who underwent endoscopic treatment for sellar or suprasellar tumors from January 2018 to March 2020. Patients’ demographics, medical histories, pathological findings, and repair outcomes were recorded. Pre- and post-operative imaging, operative reports, medical records, and operative videos were reviewed. We excluded cases if (i) post-operative pathology did not show evidence of a tumor or (ii) they were missing imaging and clinical data (**Figure 1**).

### Operation Procedure

**Figure 2:**
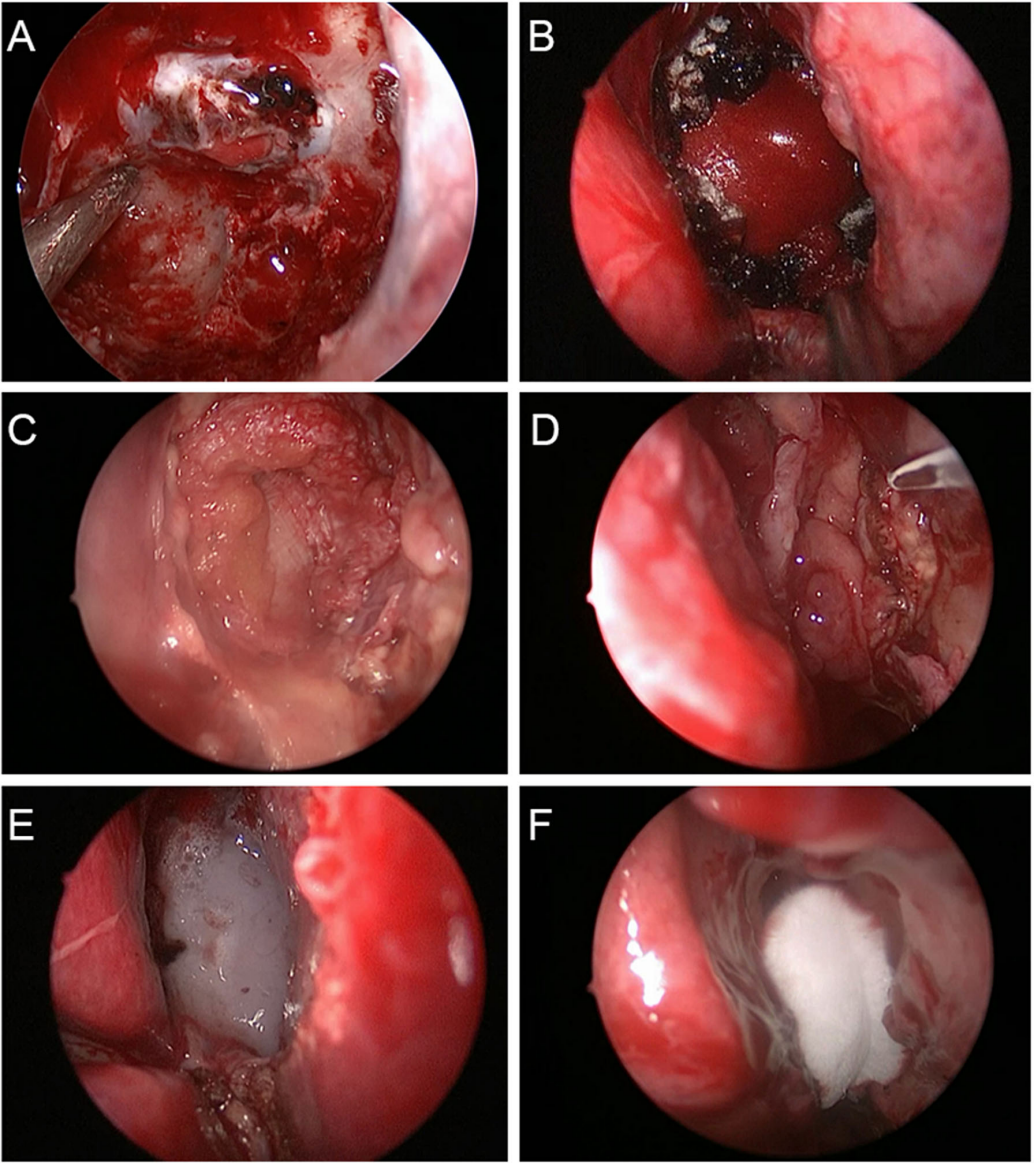
Multilayered repair procedure for cerebrospinal fluid leakage in endoscopic transsphenoidal surgery. **(A)** A piece of artificial dura mater was inserted into the subdural space. **(B)** Another piece of artificial dura mater was place in the epidural space. **(C)** Autogenous fascia lata was placed on the sellar floor for further repair. **(D)** The pediculate nasoseptal flap was used to reconstruct skull base defects. **(E)** Fibrin sealant was used for reinforcement of skull base defect repair. **(F)** The nasal cavity was filled with Nasopore dressing to strengthen the repair of skull base defects.

Endoscopic resection of sellar or suprasellar tumors was performed routinely. Most operations were performed through the right nostril. If sellar septum injury or CFL was noticed during the operation, a repair was performed *via* lumbar cistern drainage; artificial dura repair; balloon compression; or autologous tissue like fat tamponade, fascia lata, or a pediculate nasoseptal flap (**Figure 2**). Depending on the intra-operative conditions, the surgeon might choose some or all of these methods for skull base reconstruction. Regardless of the occurrence of CFL during surgery, we routinely filled the nasal cavity with an expansive sponge, which was extracted on the third day after operation. The initial head NCCT scan was performed on the first day after surgery, and the second scan was performed the first day after expansive sponge extraction.

### Calculation of NP Volume

The presence of NCCT pneumocephalus (NP) was measured on the first day after operation, and NP volume was estimated the first day after expansive sponge extraction. NP change was defined as the volume change in NP between the first day after operation and 24 h after sponge extraction, and it was divided into four categories: no NP in both NCCT measurements, NP volume decreased, NP volume remained and NP volume increased, which were abbreviated as no NP, decreased NP, unchanged NP and increased NP, respectively. The latter three categories were ascribed as NP change. NP delineation and volumetric analysis were conducted using MRIcron software (http://www.mccauslandcenter.sc.edu/mricro/mricron). The NP volume measurement method is shown in **Supplementary Figure S1**.

### Post-Operative Diagnosis of CFL

**Figure 3:**
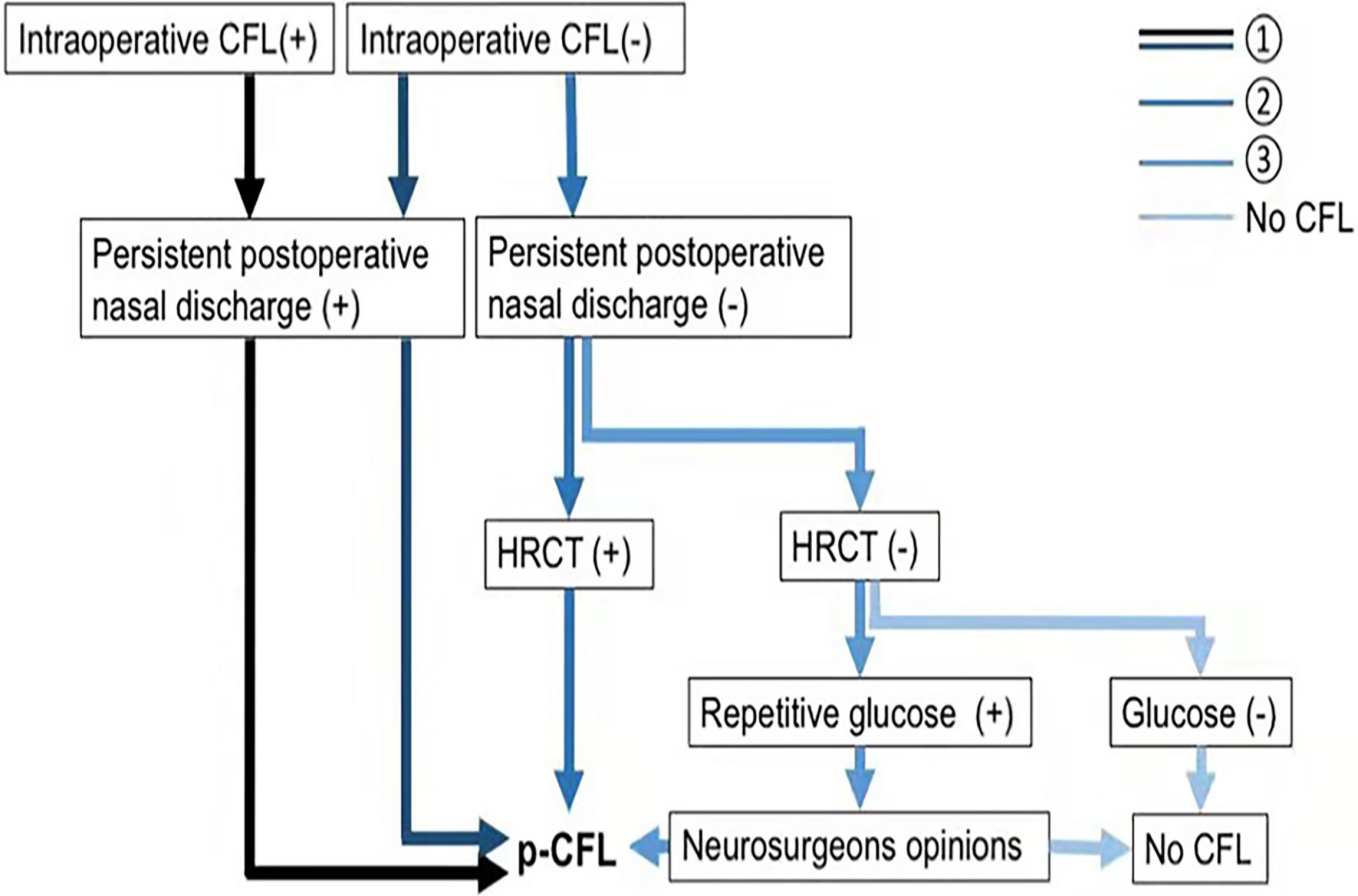
Flow chart of p-CFL diagnosis. p-CFL can be diagnosed if any of the following are met: ➀➁➂. HRCT (+) indicates a positive finding on HRCT that cerebrospinal fluid leaks through a defect, and HRCT (–) means no positive findings on HRCT. Glucose (+) indicates that glucose rhinorrhea content ≥1.7 mmol/L, and glucose (–) means that glucose rhinorrhea content <1.7 mmol/L. CFL, cerebrospinal fluid leakage; HRCT, high-resolution computer tomography; p-CFL, post-operative cerebrospinal fluid leakage.

The diagnostic flow chart is shown in **Figure 3**.

Post-operative CFL (p-CFL) was diagnosed if patients had

➀Persistent post-operative nasal discharge regardless of the occurrence of intra-operative CFL;
➁Neither intra-operative CFL nor persistent post-operative nasal discharge but positive HRCT finding (CSF leak through a defect);
➂Non-persistent nasal discharge after surgery, negative HRCT, but positive glucose test (glucose rhinorrhea content ≥1.7 mmol/L). In that case, glucose was repetitively tested, and the p-CFL diagnosis was determined by two experienced neurosurgeons (YH and WY, 5+ years’ experience in endoscopic surgery). When there were different diagnostic opinions, the neurosurgeons reached consensus after discussion.

Patients without post-operative nasal discharge and negative CSF tests were defined as non-p-CFL.

### Statistical Analysis

The characteristics of patients with and without p-CFL (i.e., p-CFL vs. non-p-CFL) are presented as mean and standard deviation (SD) for normally distributed continuous data, non-normally distributed variables are described as quartiles, and categorical variables are given as counts and percentages. Between-group differences were evaluated using Student’s t-tests or Mann–Whitney U-tests for continuous data and chi-square tests or Fisher’s exact tests for categorical variables.

Variables that were significant on univariate analysis (*p* < 0.05) were included in the binary logistic regression model. Adjusted odds ratios (ORs) with 95% confidence intervals (CIs) were calculated, and *p* < 0.05 was considered significant. Receiver operative curve (ROC) analysis was performed to test the predictive power of independent variables for the dependent variable; we calculated the areas under the ROC curve (AUC), sensitivity, and specificity. All analyses were performed after blinding the participants’ identifying information. Statistical analysis was performed using SPSS 19 (SPSS Inc., Armonk, NY, USA). ROC curve comparison analyses were conducted by MedCalc statistical software version 15 (MedCalc Software, Mariakerke, Belgium).

## Results

### Patient Characteristics

A total of 271 patients were reviewed, and we identified 253 patients who met the study criteria after excluding 18 patients for the following: (i) post-operative pathology did not show evidence of a tumor (n = 12), and (ii) incomplete imaging and clinical data from admission to discharge (n = 6). Of the 253 patients, 49.6% were female, and the median patient age was 50 years (IQR, 40–60 years).

Of all the 253 patients, 40 (15.8%) showed first-day NP after operation. After the extraction of the expansive sponge, 46 patients (18.2%) showed NP change, and 32 (12.6%) were finally confirmed with p-CFL. The median test time for p-CFL was 2 days (IQR, 1–5 days) after the removal of the expansive sponge, and 62.5% of p-CFL patients were tested beyond 2 days after the removal of the expansive sponge.

Compared with non-p-CFL group, the p-CFL group showed a significantly longer duration of hospitalization and a higher rate of post-operative infection (62.5% vs. 11.8%, χ^2^ = 48.090, *p* < 0.001) (**Supplementary Table S1**). Ten patients received the repairment operation once or more, but still two of them died because of severe intracranial infection during hospitalization.

### Factors Influencing the Occurrence of p-CFL

Compared to the non-p-CFL group, patients with p-CFL had a higher rate of intraoperative CFL, a higher use of free fat interpositional graft, lumbar cistern drainage, balloon compression, artificial dura mater implantation, and a longer duration time of operation (all *p* < 0.05). After resecting tumors, patients with p-CFL showed a higher rate of first-day NP and NP change during hospitalization, a higher occurrence of high fever and infection, and a longer duration of hospitalization (all *p* < 0.05) (**Supplementary Table S1**).

Among patients with no NP (N=207), none was found of p-CFL in both NCCT measurements. Among patients with an NP change (n = 46), 10 were in the decreased-NP subgroup who initially showed first-day NP but not on follow-up NCCT and were determined not to have p-CFL. Sixteen patients who showed increased NP between both NCCT measurements were confirmed to have p-CFL, including six patients without NP on first-day NCCT but with NP on follow-up NCCT after sponge extraction (**Figure 1**). Factors associating with NP change were tested and shown in **Supplemental Table S2**.

When adding first-day NP into the multivariate regression model, both the first-day NP (OR = 6.395, 95% CI = 2.236–18.290, p=0.001) and operative duration time (OR = 1.027, 95% CI=1.018–1.036, p<0.001) were independently associated with the occurrence of p-CFL after adjusting for pathology type (Model 1, **Supplementary Table S3**).

After adding NP change into the regression model, first-day NP was no longer independently associated with the occurrence of p-CFL. However, NP change (OR = 19.457, 95% CI = 6.292-71.373, p<0.001) and operative duration time (OR = 1.019, 95% CI=1.004-1.034, p=0.015) were independently associated with p-CFL (Model 2, **Supplementary Table S3**). In patients with NP change, decreased NP (OR = 0.133, 95%CI = 0.033-0.545, p=0.005) was significantly associated with a lower risk for having p-CFL in comparison of unchanged and increased NP.

#### Comparison of p-CFL Predictive Power Between Presence of First-Day NP and NP Change

ROC analysis showed that the AUC of first-day NP for predicting p-CFL was 0.642 (95% CI = 0.529–0.755, p=0.009), with sensitivity of 68.6% and specificity of 87.8%. The AUC of NP change for predicting p-CFL was 0.988 (95% CI = 0.977–0.998, p<0.001), with sensitivity of 100% and specificity of 93.7%. The ROC comparison showed that NP change had a significantly better predicting value than first-day NP (Z=6.451, p=0.001).

## Discussion

Diagnosis and management of p-CFL can be challenging, even for the most experienced neurosurgeons (13). Clear rhinorrhea and/or headache is common in many conditions. Those that should be considered to have CFL exhibit allergic rhinitis, common cold, vasomotor rhinitis, spontaneous intracranial hypotension, subarachnoid hemorrhage, and meningitis (18, 19). CFL can pose a serious hazard and is associated with delayed wound healing, meningitis, epidural infections, and pneumocephalus (20, 21). These complications often lead to prolonged hospitalization, reoperation, and increased healthcare costs (22–25). The most serious potential complication of CFL is meningitis (16, 26). Two patients in our study contracted *Klebsiella pneumoniae* infection and died. Thus, it is vital to find early signs that can be used to diagnose CFL. We found that both first-day post-operative NP and its volume change over time could predict p-CFL, and the latter had a higher predictive value. Compared with patients who had no NP, those with no change and increased NP had a higher risk of having p-CFL, and 100% of increased-NP patients (n = 16) were confirmed to have p-CFL.

There are two possible pathophysiologic explanations for the correlation between the presence of NP and p-CFL: the inverted bottle mechanism and the ball-valve mechanism (18, 27, 28). In the first, it is postulated that as CSF flows out of the subarachnoid space through a dural-arachnoid tear, it creates negative pressure within the subarachnoid space. The negative pressure prevents the leakage of more CSF until air enters to take its place and equilibrates the pressure differential. The ball-valve mechanism hypothesizes that air enters through a fracture next to an air-containing space (18, 27, 28). Use of a vacuum drainage system predisposes patients to pneumocephalus in the presence of CFL (18, 29, 30).

We found that the NP change after expansive sponge extraction could better predict p-CFL than the first-day NP. Previous studies considered post-operative NP as an epiphenomenon of intra-operative CSF leak which would correlate with a higher risk of p-CFL (31, 32). Head NCCT examination was routinely performed on the first day after operation when the patient’s bilateral nasal cavity was filled with an expansive sponge, and they were in a continuous supine position. Even if there was CSF leakage, it could not easily flow out of the nasal cavity. This situation also made it difficult for air to enter the brain. Therefore, if CFL is slight during and after operation, pneumocephalus is not always evident on early post-operative CT. A second CT scan was performed after sponge extraction and off-bed training for 1 day. If CFL existed at that time, CSF would be more likely to flow out than before. The reduced intracranial pressure means that air is more likely to enter the brain and causes pneumocephalus. Therefore, detecting NP volume changes can more effectively predict p-CFL than observing the NP on the first CT. However, most of the patients were discharged within 5 days of the operation, which precluded further CT dynamic observation.

In patients with first-day NP after tumor resection, the occurrence of p-CFL was lower among those with reduced NP volume compared with those with increased or unchanged NP volume. The probable cause is that pneumocephalus was gradually absorbed after the CFL resolved. Intra-operative CFL and the loss of a large amount of CSF initially leads to pneumocephalus. However, when the CFL was properly repaired with skull base reconstruction, the post-operative pneumocephalus gradually absorbed. In contrast, no change or an increase in CT pneumocephalus suggested persistent CFL. If the CFL is completely solved, the pneumocephalus will be gradually absorbed, and CT re-examination after 3 days is sufficient to observe the pneumocephalus reduction. If there is no change or there is an increase in pneumocephalus volume post-operatively, it indicates that intracranial pressure continues to decrease and a leak still exists, so the incidence of p-CFL increases.

In this study, p-CFL developed in two patients who had no pneumocephalus on the first day after the operation. Pneumocephalus was found on imaging on the fourth day after the operation, suggesting that dynamic review of head NCCT has supplementary significance for CFL evaluation. Although there was an increase in pneumocephalus volume after operation in one case, there was no CSF leakage, possibly due to excessive drainage of CSF *via* lumbar cistern drainage.

Our study was limited by the small number of patients and single-center retrospective design, so multicenter studies with larger samples sizes are needed to confirm our findings. In addition, we did not use beta-2 transferrin or beta trace protein testing to diagnose CFL because our hospital does not routinely perform these analyses. Nevertheless, glucose rhinorrhea content testing is fast, simple, inexpensive, and can be carried out in most hospitals, which could promote the wide application of our findings. An evidence-based review also pointed out that the guidance level for glucose testing was not inferior to that of beta-2 transferrin or beta trace protein testing in identifying CFL (14). Finally, the short observation time of our study was limited to the hospitalization duration, and future investigations should perform longer follow-ups.

## Conclusion

Our results show that NP change is a more convenient and effective imaging marker than first-day NP for predicting p-CFL after endoscopic sellar and suprasellar tumor resection. During dynamic NCCT observation, attention should be paid to the risk of p-CFL in patients with no change or increased NP.

## Data Availability Statement

The original contributions presented in the study are included in the article/**Supplementary Material**, further inquiries can be directed to the corresponding authors.

## Ethics Statement

This study was approved by the ethics committee of The second Affiliated Hospital of Zhejiang University, School of Medicine. The patients/participants provided their written informed consent to participate in this study.

## Author Contributions

WG, XW and SZ: drafted the manuscript. YF: acquisition of data. SZ: analysis or interpretation of data. CL, YH and WY: study concept and design. SZ and CL: help to revise the whole framework and polish the language.

## Funding

This work was supported by the National Natural Science Foundation of China (grant number 81801162), the Zhejiang Provincial Natural Science Foundation of China (grant number LGF22H090020) and the Medical Health Science and Technology Project of Zhejiang Provincial Health Commission (grant number 2022KY600).

## Conflict of Interest

The authors declare that the research was conducted in the absence of any commercial or financial relationships that could be construed as a potential conflict of interest.

## Publisher’s Note

All claims expressed in this article are solely those of the authors and do not necessarily represent those of their affiliated organizations, or those of the publisher, the editors and the reviewers. Any product that may be evaluated in this article, or claim that may be made by its manufacturer, is not guaranteed or endorsed by the publisher.

## Supplementary Material

Supplementary Figure S1 NP volume measurement on the first day and the fourth day after endoscopic transsphenoidal surgery. This is a case of 45-year-old male diagnosed with tuberculum sellae meningioma. **(A)** On the first day after operation, a small amount of CT pneumocephalus was found, which was 2.1ml. **(B)** On the fourth day after operation, CT scan showed increased pneumocephalus volume, which was 23.7ml. Cerebrospinal fluid rhinorrhea was confirmed on the sixth day after operation. The red area represents the pneumocephalus.
