# Supplementary material for: Diagnostic Value of Non-Contrast CT in Cerebrospinal Fluid Leakage After Endoscopic Transnasal Surgery for Sellar and Suprasellar Tumors
Source: Front Oncol. 2022 Jan 20;11:735778. doi: 10.3389/fonc.2021.735778 (PMC8810488; doi:10.3389/fonc.2021.735778)
Supplement: Supplementary file 1 [file Table_1.docx]

Supplementary tables

Supplementary Table S1. Comparisons of baseline and post-surgery characteristics between non-p-CFL and p-CFL

|  | Non p-CFL  (n=221) | p-CFL  (n=32) | Test value | *p* value |
| --- | --- | --- | --- | --- |
| Age, y, median (IQR) | 51 (40–60) | 48 (35.5–57) | Z=–1.065 | 0.287 |
| Female, n (%) | 112 (50.7) | 14 (43.8) | χ^2^=0.537 | 0.464 |
| Hypertension, n (%) | 56 (25.3) | 6 (18.8) | χ^2^=0.656 | 0.418 |
| Diabetes mellitus, n (%) | 18 (8.1) | 2 (6.3) | χ^2^=0.138 | 0.710 |
| **Intra-operative characteristics** | | | | |
| Trans-single nostril | 166 (75.1) | 24 (75.0) | χ^2^=0.001 | 0.989 |
| Intra-operative CFL, n (%) | 18 (8.1) | 32 (100) | χ^2^=148.732 | <0.001 |
| **Intra-operative repair measures** | | | | |
| Free fat interpositional graft | 22 (10.0) | 25 (78.1) | χ^2^=85.879 | <0.001 |
| Lumbar cistern drainage | 30 (13.6) | 24 (75.0) | χ^2^=62.822 | <0.001 |
| Balloon compression | 1 (0.5) | 4 (12.5) | χ^2^=20.943 | <0.001 |
| Artificial dura mater implantation | 153 (69.5) | 32 (100.0) | χ^2^=13.275 | <0.001 |
| Operation duration, min, median (IQR) | 105 (80–140) | 222 (175–255) | Z=–6.941 | <0.001 |
| Tumor size, mm | 23 (18–30) | 26 (20–34) | Z=–1.676 | 0.094 |
| Pathology types, n (%) |  |  | χ^2^=38.975 | <0.001 |
| Non-functional tumor | 84 (38.0) | 4 (12.5) |  |  |
| Functional adenoma | 58 (26.2) | 6 (18.8) |  |  |
| Meningioma | 2 (0.9) | 9 (28.1) |  |  |
| Craniopharyngioma | 12 (5.4) | 10 (31.3) |  |  |
| Others^*^ | 56 (25.3) | 12 (37.5) |  |  |
| **Post-operative characteristics** | | | | |
| First-day NP, n (%) | 27 (12.2) | 13 (40.6) | χ^2^=16.947 | <0.001 |
| No NP | 207 (93.7) | 0 (0) | χ^2^=188.637 | <0.001 |
| NP change^&^, n (%) |  |  | χ^2^=12.413 | 0.002 |
| Decreased | 10 (4.5) | 8 (25.0) |  |  |
| Unchanged | 4 (1.8) | 8 (25.0) |  |  |
| Increased | 0 (0) | 16 (50) |  |  |
| High fever, n (%) | 26 (11.8) | 20 (62.5) | χ^2^=48.368 | <0.001 |
| Infection, n (%) | 26 (11.8) | 20 (62.5) | χ^2^=48.090 | <0.001 |
| Residual tumor, n (%) | 109 (49.3) | 12 (37.5) | χ^2^=1.565 | 0.211 |
| Long-term hypopituitarism, n (%) | 92 (41.6) | 13 (40.6) | χ^2^=0.012 | 0.914 |
| Hospitalization duration, days | 5(4–7) | 17(11–24) | χ^2^=–7.883 | <0.001 |

*Others in the pathology section include growth hormone adenomas, prolactin adenomas, adrenocorticotropic hormone adenomas, thyroid-stimulating hormone adenomas, gonadotroph adenomas, and mixed tumors.

^&^means that this analysis was only performed in NP change group (n=46).

CFL, cerebrospinal fluid leakage; IQR, interquartile range; NP: non-contrast computed tomography pneumocephalus.

Supplementary Table S2. Univariate analysis for pre- and intra-operative factors relates to NP change

|  | No NP | Decreased NP | Unchanged NP | Increased NP | Test value | *P* value |
| --- | --- | --- | --- | --- | --- | --- |
|  | (n=207) | (n=18) | (n=12) | (n=16) |  |  |
| Age, y, median (IQR) | 51 (40-60) | 48 (44-53) | 43 (24-47) | 53 (34-58) | F=2.111 | 0.099 |
| Female, n (%) | 105 (50.7) | 9 (50) | 6 (50) | 6 (37.5) | χ^2^=1.040 | 0.792 |
| Hypertension, n (%) | 55 (26.6) | 5 (27.8) | 0 (0) | 2 (12.5) | χ^2^=5.723 | 0.126 |
| Diabetes mellitus, n (%) | 17 (8.2） | 3 (16.7) | 0 (0) | 0 (0) | χ^2^=4.328 | 0.228 |
| Trans-single nostril | 157 (75.8) | 13 (72.2) | 7 (58.3) | 13 (81.3) | χ^2^=2.269 | 0.519 |
| Lumbar cistern drainage | 28 (13.5) | 4 (22.2) | 9 (75) | 13 (81.3) | χ^2^=62.324 | <0.001 |
| Operation duration, min, median (IQR) | 105 (75-140) | 148 (85-184) | 180 (145-254) | 255 (184-271) | F=32.976 | <0.001 |
| Tumor size, mm | 25.1±10.3 | 24.1±8.4 | 25.8±15.6 | 30.6±11.2 | F=1.442 | 0.231 |
| Pathology types, n (%) | |  |  |  | χ^2^=31.917 | 0.001 |
| Non-functional tumor | 77 (37.2) | 4 (22.2) | 5 (41.7) | 2 (12.5) |  |  |
| Functional adenoma | 57 (27.5) | 3 (16.7) | 1 (8.3) | 3 (18.8) |  |  |
| Meningioma | 12 (5.8) | 3 (16.7) | 2 (16.7) | 5 (31.3) |  |  |
| Craniopharyngioma | 30 (14.5) | 2 (11.1) | 0 (0) | 0 (0) |  |  |
| Others | 31 (15.0) | 6 (33.3) | 4 (33.3) | 6 (37.5) |  |  |

CFL, cerebrospinal fluid leakage; IQR, interquartile range; NP: non-contrast CT pneumocephalus.

Supplementary Table S3. Multivariate regression analysis for variables associating with p-CFL

| Model 1 | OR | 95% CI | *p* value |
| --- | --- | --- | --- |
| First-day NP | 6.395 | 2.236-18.290 | 0.001 |
| Operative duration time | 1.027 | 1.018–1.036 | <0.001 |
| Pathology types | 1.051 | 0.760–1.453 | 0.765 |
| Model 2 | OR | 95% CI | *p* value |
| First-day NP | 2.799 | 0.523-14.974 | 0.229 |
| NP change | 19.457 | 6.095-62.107 | <0.001 |
| Operative duration time | 1.019 | 1.004–1.034 | 0.015 |
| Pathology types | 1.370 | 0.773–2.427 | 0.281 |

CFL, cerebrospinal fluid leakage; OR, odds ratio; CI, confidence interval; NP: non-contrast computed tomography pneumocephalus.
