# Supplementary figures and images for: Diagnostic Value of Non-Contrast CT in Cerebrospinal Fluid Leakage After Endoscopic Transnasal Surgery for Sellar and Suprasellar Tumors
Source: Front Oncol. 2022 Jan 20;11:735778. doi: 10.3389/fonc.2021.735778 (PMC8810488; doi:10.3389/fonc.2021.735778)

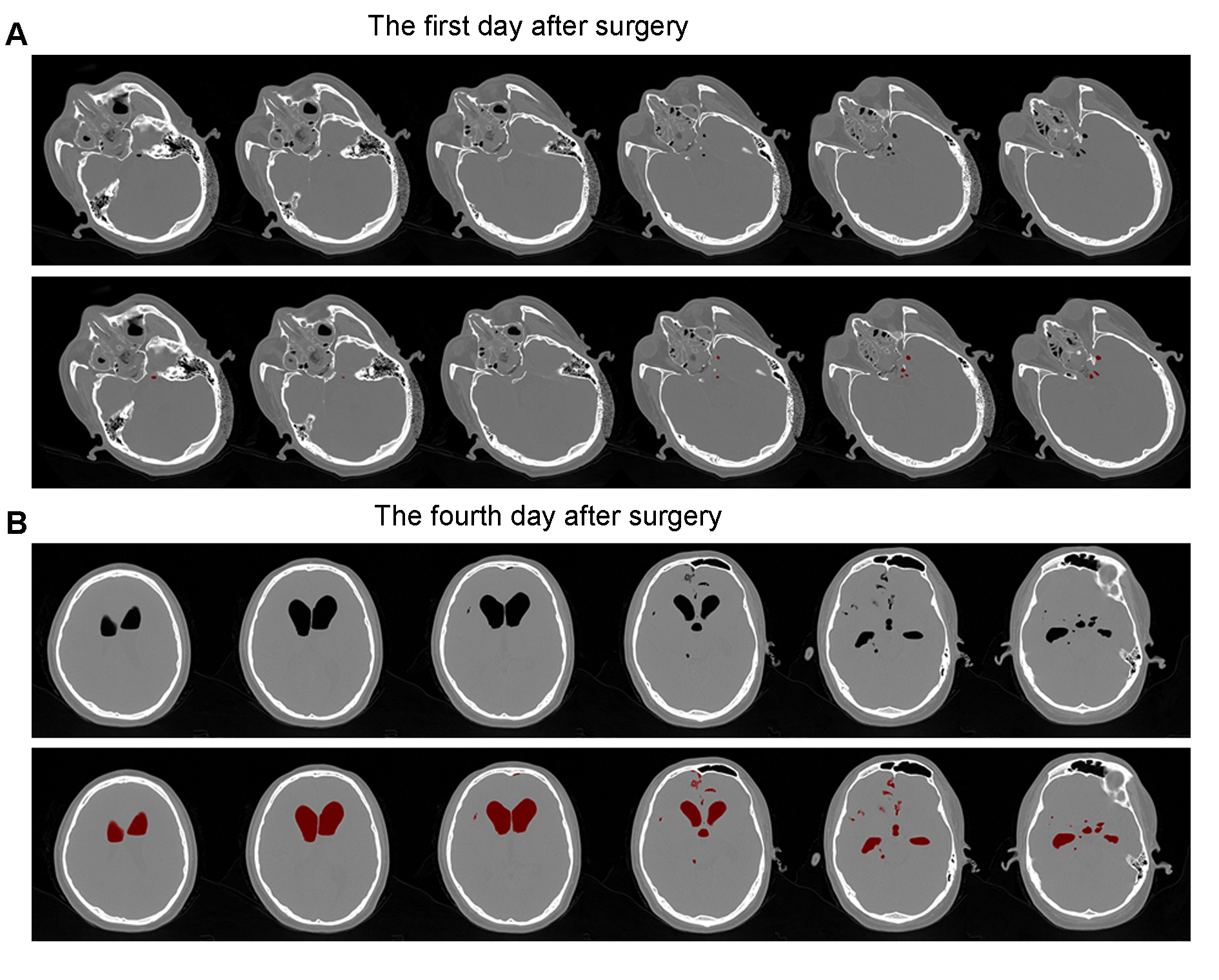

Supplement: Supplementary Figure S1 — NP volume measurement on the first day and the fourth day after endoscopic transsphenoidal surgery. This is a case of 45-year-old male diagnosed with tuberculum sellae meningioma. (A) On the first day after operation, a small amount of CT pneumocephalus was found, which was 2.1ml. (B) On the fourth day after operation, CT scan showed increased pneumocephalus volume, which was 23.7ml. Cerebrospinal fluid rhinorrhea was confirmed on the sixth day after operation. The red area represents the pneumocephalus. [file Image_1.jpeg]
